# Fluorine-18 Prostate-Specific Membrane Antigen–1007 PET/CT vs Multiparametric MRI for Locoregional Staging of Prostate Cancer

**DOI:** 10.1001/jamaoncol.2024.3196

**Published:** 2024-07-01

**Authors:** Nikhile Mookerji, Tyler Pfanner, Amaris Hui, Guocheng Huang, Patrick Albers, Rohan Mittal, Stacey Broomfield, Lucas Dean, Blair St. Martin, Niels-Erik Jacobsen, Howard Evans, Yuan Gao, Ryan Hung, Jonathan Abele, Peter Dromparis, Joema Felipe Lima, Tarek Bismar, Evangelos Michelakis, Gopinath Sutendra, Frank Wuest, Wendy Tu, Benjamin A. Adam, Christopher Fung, Alexander Tamm, Adam Kinnaird

**Affiliations:** 1 Division of Urology, Department of Surgery, University of Alberta, Edmonton, Alberta, Canada; 2 Department of Radiology & Diagnostic Imaging, University of Alberta, Edmonton, Alberta, Canada; 3 Department of Laboratory Medicine & Pathology, University of Alberta, Edmonton, Alberta, Canada; 4 Alberta Centre for Urologic Research and Excellence (ACURE), Alberta, Canada; 5 Department of Pathology & Laboratory Medicine, University of Calgary, Calgary, Alberta, Canada; 6 Alberta Prostate Cancer Research Initiative (APCaRI), Alberta, Canada; 7 Department of Medicine, University of Alberta, Edmonton, Alberta, Canada; 8 Cancer Research Institute of Northern Alberta (CRINA), Edmonton, Alberta, Canada; 9 Department of Oncology, University of Alberta, Edmonton, Alberta, Canada

## Abstract

**Importance:**

Prostate-specific membrane antigen (PSMA) demonstrates overexpression in prostate cancer and correlates with tumor aggressiveness. PSMA positron emission tomography (PET) is superior to conventional imaging for the metastatic staging of prostate cancer per current research but studies of second-generation PSMA PET radioligands for locoregional staging are limited.

**Objective:**

To determine the accuracy of fluorine-18 PSMA-1007 PET/computed tomography (^18^F-PSMA-1007 PET/CT) compared to multiparametric magnetic resonance imaging (MRI) in the primary locoregional staging of intermediate-risk and high-risk prostate cancers.

**Design, Setting, and Participants:**

The Next Generation Trial was a phase 2 prospective validating paired cohort study assessing the accuracy of ^18^F-PSMA-1007 PET/CT and MRI for locoregional staging of prostate cancer, with results of histopathologic examination as the reference standard comparator. Radiologists, nuclear medicine physicians, and pathologists were blinded to preoperative clinical, pathology, and imaging data. Patients underwent all imaging studies and radical prostatectomies at 2 tertiary care hospitals in Alberta, Canada. Eligible participants included men with intermediate-risk or high-risk prostate cancer who consented to radical prostatectomy. Participants who underwent radical prostatectomy were included in the final analysis. Patients were recruited between March 2022 and June 2023, and data analysis occurred between July 2023 and December 2023.

**Exposures:**

All participants underwent both ^18^F-PSMA-1007 PET/CT and MRI within 2 weeks of one another and before radical prostatectomy.

**Main Outcomes and Measures:**

The primary outcome was the correct identification of the prostate cancer tumor stage by each imaging test. The secondary outcomes were correct identification of the dominant nodule, laterality, extracapsular extension, and seminal vesical invasion.

**Results:**

Of 150 eligible men with prostate cancer, 134 patients ultimately underwent radical prostatectomy (mean [SD] age at prostatectomy, 62.0 [5.7] years). PSMA PET was superior to MRI for the accurate identification of the final pathological tumor stage (61 [45%] vs 38 [28%]; *P* = .003). PSMA PET was also superior to MRI for the correct identification of the dominant nodule (126 [94%] vs 112 [83%]; *P* = .01), laterality (86 [64%] vs 60 [44%]; *P* = .001), and extracapsular extension (100 [75%] vs 84 [63%]; *P* = .01), but not for seminal vesicle invasion (122 [91%] vs 115 [85%]; *P* = .07).

**Conclusions and Relevance:**

In this phase 2 prospective validating paired cohort study, ^18^F-PSMA-1007 PET/CT was superior to MRI for the locoregional staging of prostate cancer. These findings support PSMA PET in the preoperative workflow of intermediate-risk and high-risk tumors.

## Key Points

**Question:**

Is fluorine-18 prostate-specific membrane antigen–1007 positron emission tomography/computed tomography (^18^F-PSMA-1007 PET/CT) superior to multiparametric magnetic resonance imaging (MRI) for the locoregional primary staging of intermediate-risk and high-risk prostate cancers before treatment?

**Findings:**

In this phase 2 prospective validating paired cohort study of 134 men undergoing radical prostatectomy, ^18^F-PSMA-1007 PET/CT correctly identified the final pathological tumor stage in 61 men (45%) compared to 38 men (28%) with multiparametric MRI, a statistically significant difference.

**Meaning:**

These findings support the use of ^18^F-PSMA-1007 PET/CT in the pretreatment staging of intermediate-risk and high-risk prostate tumors, favoring its accuracy over multiparametric MRI.

## Teaser

This study assesses the accuracy of fluorine-18 prostate-specific membrane antigen–1007 positron emission tomography (PET)/computed tomography (CT) compared to multiparametric magnetic resonance imaging (MRI) in locoregional staging of prostate cancer.

## Introduction

The diagnosis and management of intermediate-risk and high-risk prostate cancer are increasingly informed by advances in diagnostic imaging.^1^ Current guidelines recommend the use of multiparametric magnetic resonance imaging (MRI) to assist with diagnosis and locoregional staging of prostate cancer before radical prostatectomy.^2,3,4^ According to research studies, prostate-specific membrane antigen positron emission tomography (PSMA PET) is superior to conventional imaging (computed tomography [CT] and bone scan) for nodal and metastatic staging.^5,6,7,8,9^ For several factors, such as urinary accumulation of PSMA radioligands excreted by the kidneys, low-grade background prostatic activity, and lower resolution of PET compared to MRI, the accuracy of prostate cancer tumor staging has been limited.

Fluorine-18 PSMA-1007 PET/CT (^18^F-PSMA-1007 PET/CT) is a PSMA radioligand that features high PSMA affinity, higher spatial resolution, and longer half-life than gallium 68 (^68^Ga) radioligands, as well as minimal urinary excretion.^10^ Case series suggest that ^18^F-PSMA-1007 may provide accurate detection of intraprostatic tumor nodules and have a role in primary staging.^11,12^

Given the limited evidence for ^18^F-PSMA-1007 compared with MRI in the locoregional staging of prostate cancer, we conducted a phase 2 prospective validating paired cohort study (Oxford Level of Evidence 1B) in men undergoing radical prostatectomy for intermediate-risk and high-risk prostate cancer.

## Methods

### Study Design

The Next Generation Trial was an investigator-initiated phase 2 prospective validating paired cohort study conducted at 2 hospitals in Alberta, Canada. The trial was registered at ClinicalTrials.gov (NCT05141760). The trial received approval from Health Canada and the Health Research Ethics Board of Alberta Cancer Committee. The trial was monitored by the Northern Alberta Clinical Trials and Research Centre. This study was reported following the Standards for Reporting of Diagnostic Accuracy (STARD) reporting guideline.^13^ Patients were recruited between March 2022 and June 2023, and data analysis occurred between July 2023 and December 2023.

### Study Participants

Adult patients with a diagnosis of intermediate-risk or high-risk prostate cancer considering robot-assisted laparoscopic radical prostatectomy with bilateral pelvic lymph node dissection were eligible for enrollment. Patients were assessed for eligibility between March 2022 and June 2023. Patients were excluded if they were unable to give informed consent, weighed more than 250 kg due to the weight limitation of scanners, were unable to lie flat for 30 minutes to complete the PET or MRI imaging, had severe claustrophobia precluding image acquisition, lacked intravenous access, had non-MRI–compatible pacemaker or hardware, had an estimated glomerular filtration rate (eGFR) of less than 40 mL/min/1.73 m^2^, a history of a severe reaction to MRI contrast, or had a history of prior androgen deprivation therapy. All participants provided written informed consent.

### Trial Procedure

Participants underwent both an ^18^F-PSMA-1007 PET/CT and multiparametric MRI within 14 days of one another and at least 5 days before surgery. Radiologists, nuclear medicine physicians, and pathologists were blinded to all other imaging, biopsy, and clinical data for each participant. Multiparametric MRI sequences were obtained using 1 of 2 magnets (Magnetom Skyra or Vida 3 Tesla [Siemens]) and were interpreted using the Prostate Imaging Reporting and Data System (PI-RADS) version 2.1 criteria.^14^ MRI interpretations were performed by consensus reading by at least 2 radiologists (fellowship-trained body radiologists who had each performed at least 1500 previous prostate MRI interpretations). Sequences from ^18^F-PSMA-1007 PET/CT were obtained using 1 of 2 PET scanners (Discovery MI PET/CT [GE HealthCare] or Biograph Vision PET/CT [Siemens]) and were interpreted using modified Prostate Cancer Molecular Imaging Standardized Evaluation (PROMISE) guidelines (excluding MRI PI-RADS class as the nuclear physicians were blinded to the MRI).^12^ Grade 2 or higher nodular foci of activity were considered malignant nodules. Nodular grade 1 activity that was still greater than blood pool and reactive nodes with enhancement 10 Hounsfield Units between the arterial and venous phase or with contiguous extraprostatic extension were considered malignant nodules.^3^ To measure nodule characteristics and determine extraprostatic extension, using Oasis software (Segami Corporation), a tumoral volume of interest was defined by using a spherical region of interest around the suspected malignant nodule and then having the software automatically identify 1-mm voxel sizes that were greater than 30% maximum standardized uptake value for grade 2 or 3 nodules and 50% to 65% maximum standardized uptake value for grade 1 nodules.^11^ Extraprostatic extension was defined as the volume of interest extending more than 1 mm into the periprostatic fat (T3a) or if the volume of interest demonstrates contiguous linear extension from the nodule into the neurovascular bundle, seminal vesicles (T3b), or adjacent organs (T4). PET interpretations were performed by consensus reading by a nuclear medicine physician and senior combined radiology and nuclear medicine resident (fewer than 100 previous ^18^F-PSMA-1007 interpretations collectively). Participants underwent standard robot-assisted laparoscopic radical prostatectomy with bilateral pelvic lymph node dissection. Final pathology was centrally reviewed, and standardized reports containing overall pathological information as well as data for each cancer nodule were collected.^15^ Schematics drawn on a 38-segment prostate schematic were also used for analysis.

### Protocol for Multiparametric MRI

Following manual shim and application of saturation bands across the anterior abdomen, overlying sigmoid colon, and the rectum, magnetic resonance sequences acquired through the pelvis including initial localizer sequences, wide field of view axial T2, high resolution axial/coronal/sagittal T2 of the prostate, axial diffusion-weighted imaging calculated to b1600 with an associated apparent diffusion coefficient map, and axial T1 volumetric interpolated breath-hold examination before and after gadolinium contrast. Postgadolinium T1 volumetric interpolated breath-hold examination sequences were acquired every 11 seconds for a total of 132 seconds. Gadolinium was injected by hand over 10 seconds via a 20- to 22-gauge needle with a dose of 0.1 mL/kg and a subsequent 20-mL flush of 0.9% normal saline.

### Protocol for ^18^F-PSMA-1007 PET/CT

Four megabecquerel/kg (1 becquerel = 2.7 × 10^−11^ curie [Ci]), with a minimum of 2 megabecquerel/kg and maximum of 400 megabecquerel, of ^18^F-PSMA-1007 were injected intravenously with the patients reclined in a standard PET uptake room. After a 2-hour uptake (range, 100 to 140 minutes from injection), patients were transferred to the PET scanner table. Patients were asked to void completely immediately prior to the scan. A standard PET acquisition was performed, beginning at the vertex and progressing downward to the toes. This was completed at 4 minutes per bed position, or equivalent bed movement speed depending on the scanner used and body part imaged. Over the legs, 2 minutes per bed position was conducted to minimize the time between PET and the subsequently acquired CT. The multiphase diagnostic CT scan of the same anatomic region was acquired for attenuation correction and diagnostic interpretation, with the administration of intravenous contrast, unless contraindicated. The serum creatinine (within 30 days of the study) and calculated eGFR were reviewed. If unavailable, serum creatinine was ascertained with an i-Stat 1 analyzer (Abbott Point of Care Inc) immediately before the scan. Patients were screened with a verbal questionnaire regarding history of intravenous iodinated contrast allergy. Intravenous contrast was administered for patients with eGFR of 40 mL/min/1.73 m^2^ or more and no history of anaphylactic allergic reaction to contrast. Iohexol (Omnipaque 350 [GE HealthCare]), 100 mL, was injected intravenously via a 20- to 22-gauge needle at a rate of 3.5 mL/s followed by 25 mL of normal saline at a rate of 3 mL/s. Helical CT images were obtained from the vertex to the toes at 25 seconds after injection and from the top of the diaphragm to the symphysis pubis at 80 seconds after injection. CT acquisition parameters for all CT scans were as follows: 120 kilovolts, 250 to 300 milliampere-seconds, and 50- to 70-cm field of view. Pitch, rotation speed, and slice thickness may vary slightly by scanner and body part imaged. The reconstructed PET images and correlative CT images were reviewed using Segami Oasis platform workstations (Segami Corporation).

### Outcomes

The primary outcome was correct identification of the prostate cancer tumor stage.^15^ The secondary outcomes were correct identification of the dominant nodule, laterality, extracapsular extension, and seminal vesicle invasion.

### Statistical Analysis

The study was designed to have 90% power to detect a 10% superiority in the correct identification of the prostate cancer tumor stage by PSMA PET. Accounting for an estimated attrition rate of approximately 10% to 15%, 150 participants were required. The McNemar test with Holm-Bonferroni correction was used to compare accuracy between the 2 imaging tests. Two-sided *P* values of .05 or less indicated statistical significance. Analyses were performed using STATA software, version 18 (StataCorp).

## Results

### Patient Characteristics

Between March 2022 and June 2023, 275 patients were assessed for eligibility (Figure). A total of 150 men with prostate cancer were enrolled and had both scans (^18^F-PSMA-1007 PET/CT and multiparametric MRI) completed at 1 of 2 tertiary care hospitals in Alberta, Canada. Sixteen patients were excluded after their scans: 10 patients due to opting for radiation therapy, 4 patients for having metastasis detected on PSMA PET imaging, and 2 patients opting for no further treatment. Of the 134 patients in the final analysis, the mean (SD) age at prostatectomy was 62.0 (5.7) years. Clinical and pathological characteristics are listed in Table 1 and eTable 1 in Supplement 1. A total of 127 patients (95%) had Gleason grade group 2 or 3 and 65 (49%) had extraprostatic extension (T3a or T3b). A total of 118 patients (88%) received a PI-RADS score of 3 or greater, and 134 (100%) had nodules meeting the modified PROMISE criteria described previously.

**Figure.**
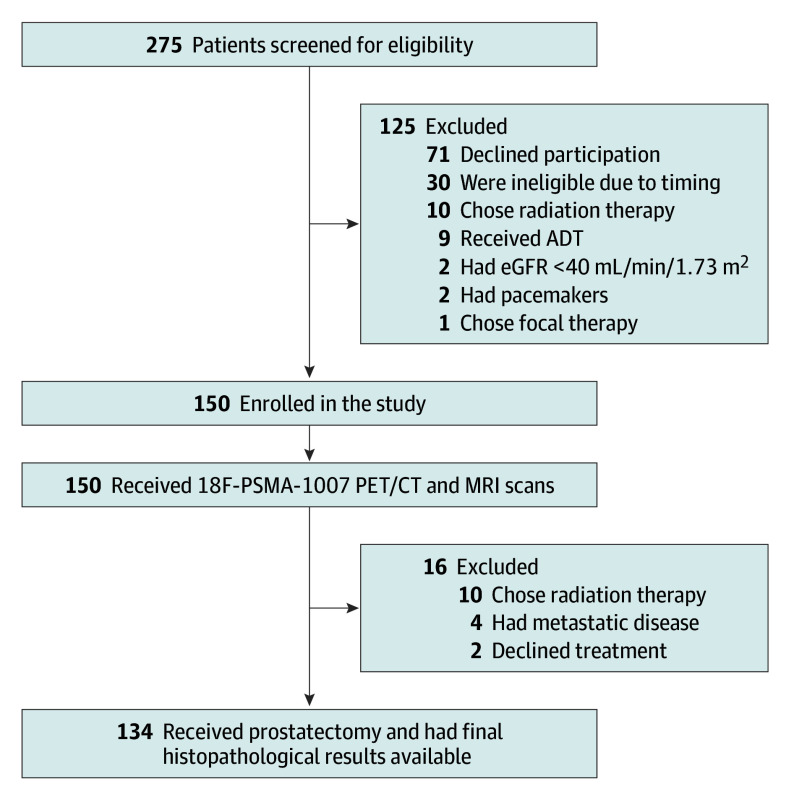
Flow Diagram of Study Design and Participants This diagram shows the flow of patients through the study from 275 patients assessed for eligibility to 150 patients enrolled and 134 patients with final analyzable data. ADT indicates androgen deprivation therapy; eGFR, estimated glomerular filtration rate; MRI, magnetic resonance imaging; PET/CT, positron emission tomography/computed tomography; and PSMA, prostate-specific membrane antigen.

**Table 1.** Clinical and Pathological Characteristics at Time of Radical Prostatectomy

Characteristic	No. (%) (N = 134)
Age at prostatectomy, mean (SD), y	62.0 (5.7)
Preoperative PSA, median (IQR), ng/mL	7.8 (6.7-10.9)
Final pathology Gleason grade group	
Benign	1 (1)
1	0
2	94 (70)
3	33 (25)
4	1 (1)
5	5 (4)
Final pathological tumor stage	
T0	1 (1)
T2a	5 (4)
T2b	4 (3)
T2c	59 (44)
T3a	44 (33)
T3b	21 (16)
T4	0
Final pathological nodule stage	
N0	130 (97)
N1	4 (3)
Pathological prostate volume, median (IQR), cc	39 (31-47)
MRI PI-RADS score,	
≤2	16 (12)
3	0
4	45 (34)
5	73 (54)
PSMA PET modified PROMISE scores	
0	0
1	54 (40)
2	42 (31)
3	38 (28)
Dominant nodule pathological volume, median (IQR), cc	6.2 (3-13)
Dominant nodule maximum SUV on PSMA PET, median (IQR)	16.9 (10-27)

Abbreviations: MRI, magnetic resonance imaging; PI-RADS, Prostate Imaging Reporting and Data System; PROMISE, Prostate Cancer Molecular Imaging Standardized Evaluation; PSA, prostate-specific antigen; PSMA PET, prostate-specific membrane antigen positron emission tomography; SUV, standardized uptake value.

### Primary Outcome

PSMA PET was superior to MRI for the accurate identification of the pathological tumor stage (61 [45%] vs 38 [28%]; *P* = .003) (Table 2). PSMA PET and MRI underestimated the tumor stage in 55 patients (41%) and 86 patients (64%), respectively (eTable 2 in Supplement 1).

**Table 2.** Correct Identification of Pathological Parameters by Preoperative ^18^F-PSMA-1007 PET/CT and MRI

Pathological variable	Correct identification, No. (%) [95% CI] (N = 134)		
	MRI	^18^F-PSMA-1007 PET/CT	*P* value
Final pathological tumor stage	38 (28) [21-37]	61 (45) [36-54]	.003
Dominant nodule	112 (83) [75-89]	126 (94) [88-97]	.01
Laterality	60 (44) [36-53]	86 (64) [55-72]	.001
Extracapsular extension	84 (63) [54-71]	100 (75) [67-82]	.01
Seminal vesical invasion	115 (85) [78-90]	122 (91) [84-95]	.07

Abbreviations: ^18^F-PSMA-1007 PET/CT, fluorine-18 prostate-specific membrane antigen–1007 positron emission tomography/computed tomography; MRI, magnetic resonance imaging; PSMA PET, prostate-specific membrane antigen positron emission tomography.

### Secondary Outcomes and Safety

PSMA PET was superior to MRI for the correct identification of the dominant nodule (126 [94%] vs 112 [83%]; *P* = .01), laterality (ie, unilateral or bilateral, 86 [64%] vs 60 [44%]; *P* = .001), and extracapsular extension (100 [75%] vs 84 [63%]; *P* = .01), but not statistically significantly for seminal vesicle invasion (122 [91%] vs 115 [85%]; *P* = .07). Sensitivity for extracapsular extension was 58% (95% CI, 45%-70%) and 33% (95% CI, 22%-46%) for PSMA PET and MRI, respectively (Table 3). Specificity for extracapsular extension was 90% (95% CI, 80%-96%) and 90% (95% CI, 80%-96%) for PSMA PET and MRI, respectively. Sensitivity for seminal vesicle invasion was 57% (95% CI, 34%-77%) and 33% (95% CI, 15%-57%) for PSMA PET and MRI, respectively. Specificity for seminal vesicle invasion was 97% (95% CI, 92%-99%) and 96% (95% CI, 89%-98%) for PSMA PET and MRI, respectively. Sensitivity for lymph node metastasis was 50% (95% CI, 9%-91%) and 25% (95% CI, 1%-78%) for PSMA PET and MRI, respectively. Specificity for lymph node metastasis was 98% (95% CI, 94%-100%) and 100% (95% CI, 96%-100%) for PSMA PET and MRI, respectively (eTable 3 in Supplement 1). On a per-tumor nodule analysis, PSMA PET detected more of the Gleason grade group 2 or greater nodules than MRI (201 of 231 [87%] vs 143 of 231 [62%], respectively; *P* < .001) (eTable 4 in Supplement 1). Similarly, PSMA PET detected more Gleason grade group 1 nodules than MRI (16 of 55 [29%] vs 6 of 55 [11%], respectively; *P* = .02). No patient experienced an adverse event related to an imaging study in this trial.

**Table 3.** Diagnostic Accuracy of ^18^F-PSMA-1007 PET/CT and MRI for Laterality of Disease, Extracapsular Extension, and Seminal Vesicle Invasion

Parameter	% (95% CI)		
	MRI	^18^F-PSMA-1007 PET/CT	
**Laterality**			
Sensitivity	39 (31-49)		69 (60-77)
Specificity	93 (64-100)		27 (9-55)
PPV	98 (88-100)		88 (79-94)
NPV	15 (9-25)		10 (3-24)
**Extracapsular extension**			
Sensitivity	33 (22-46)		58 (45-70)
Specificity	90 (80-96)		90 (80-96)
PPV	75 (55-89)		84 (69-93)
NPV	59 (49-69)		70 (59-79)
**Seminal vesicle invasion**			
Sensitivity	33 (15-57)		57 (34-77)
Specificity	96 (89-98)		97 (92-99)
PPV	58 (29-84)		80 (51-95)
NPV	89 (81-93)		92 (86-96)

Abbreviations: ^18^F-PSMA-1007 PET/CT, fluorine-18 prostate-specific membrane antigen–1007 positron emission tomography/computed tomography; MRI, magnetic resonance imaging; NPV, negative predictive value; PPV, positive predictive value.

## Discussion

The results of this prospective validating paired cohort study suggest that ^18^F-PSMA-1007 PET/CT is superior to multiparametric MRI for the locoregional tumor staging of intermediate-risk and high-risk prostate cancers in men undergoing radical prostatectomy. Although the localization of the dominant tumor nodules was identified in most patients by both imaging techniques, accurate tumor staging was observed in only approximately half of PSMA PET scans and one-quarter of MRI scans. One explanation for this relatively low degree of overall accuracy is that blinding the radiologists and nuclear medicine physicians to biopsy results and other imaging data impaired the ability to make a diagnosis that would be possible outside of the trial in a standard clinical setting. Furthermore, the additive or synergistic effect of the combination of the 2 imaging tests by the same reader has been demonstrated with ^68^Ga–PSMA HBED-CC PET/MRI but, to our knowledge, remains to be studied with ^18^F-PSMA-1007.^16^

In this study, PSMA PET outperformed MRI in detecting extracapsular extension. In keeping with previous publications, MRI had high specificity but poor sensitivity for extracapsular extension.^17^ PSMA PET showed improved sensitivity and remained highly specific. The finding that PSMA PET better diagnosed extracapsular extension due to the presumed higher spatial resolution of MRI imaging compared to PSMA PET was an unanticipated finding and may be a unique feature of the ^18^F-PSMA-1007 radioligand as this was not previously observed with first-generation gallium-based radioligands.^18^

High-risk features such as extracapsular extension are critical to identify on imaging before radical prostatectomy as their presence alters the surgical approach. For instance, preoperative knowledge of extracapsular extension may alter the decision to perform nerve-sparing techniques on the side with anticipated tumor extension.^19^ Similarly, it may alter the extent of margin that is sought during pericapsular dissection. In this study, nearly two-thirds of patients were under-staged when using MRI compared to approximately 40% of men when using PSMA PET.

PSMA PET more accurately determined unilateral vs bilateral disease than MRI. These findings are important for novel treatments for patients with prostate cancer, such as focal therapies. The premise of focal therapy is to ablate only the side of the prostate where the cancer is present, thereby sparing the contralateral side from harmful treatment effects. Therefore, the presence of bilateral disease is a contraindication to focal therapy. In this study, PSMA PET had an approximately 20% improvement over MRI in decerning unilateral and bilateral tumors. Furthermore, PSMA PET demonstrated increased detection of all Gleason grade group 2 or higher tumor nodules (ie, nodules requiring treatment). Therefore, PSMA-directed focal therapy may be an approach for future trials.

This trial used the *American Joint Committee on Cancer (AJCC) Staging Manual, seventh edition* to improve the ability to assess imaging accuracy for organ-confined tumors.^15^ This is because the seventh edition breaks the final pathology staging into T2a, T2b, and T2c, whereas more recent editions combine those groups into a singular organ-confined T2, which limits the ability to discern certain features such as laterality.^20^

Determination of differences in the specificity and negative predictive value for PSMA PET compared to MRI is limited in this trial as all patients were diagnosed with prostate cancer before imaging (ie, the theoretical number of true negative patients is zero). Interestingly, the first patient to enter the trial was found to be T0 at final pathology after radical prostatectomy despite a preoperative diagnosis of Gleason grade group 4 prostate cancer.^21^ Notably, this patient had a negative result on MRI yet had a positive grade for cancer on PSMA PET.

The safety profiles of ^18^F-PSMA-1007 PET/CT and MRI appeared to be similar as none of the 150 men who underwent both imaging tests experienced an adverse event. This is an important finding if PSMA PET is to be expanded to more indications including preoperative imaging of intermediate-risk prostate cancers. Furthermore, the cost-effectiveness will need to be assessed. Based on our findings and those previously published for metastatic staging, the adoption of a single preoperative PSMA PET scan may potentially replace the trio of preoperative conventional imaging tests currently ordered (MRI, CT abdomen and pelvis, and bone scan) in certain patients with intermediate-risk and high-risk prostate cancer.

### Limitations

This study had limitations, which included the inability to use whole-mount specimens for pathological analysis. In addition, the absence of a standardized extended template lymph node dissection at the time of radical prostatectomy limited our ability to determine the accuracy of nodal staging for each imaging type.

## Conclusions

In this prospective paired cohort study, ^18^F-PSMA-1007 PET/CT provided improved accuracy compared with multiparametric MRI for prostate cancer tumor staging before radical treatment. Future research should address the accuracy of combining PET/CT and MRI as well as cost effectiveness of PET/CT for the primary staging of men with prostate cancer.

## Notes

Supplement 1. eTable 1. Preoperative biopsy characteristics eTable 2. Over staging and under staging of final pathological T staging by multiparametric MRI and ^18^F-PSMA-1007 PET/CT eTable 3. Diagnostic accuracy of ^18^F-PSMA-1007 PET/CT and MRI for lymph node metastasis eTable 4. Nodule level detection by multiparametric MRI and ^18^F-PSMA-1007 PET/CT Stratified by Gleason grade group of individual prostate cancer nodules

Supplement 2. Data sharing statement
