# Supplementary material for: Fluorine-18 Prostate-Specific Membrane Antigen–1007 PET/CT vs Multiparametric MRI for Locoregional Staging of Prostate Cancer
Source: JAMA Oncol. 2024 Jul 1;10(8):1097–103. doi: 10.1001/jamaoncol.2024.3196 (PMC11217889; doi:10.1001/jamaoncol.2024.3196)
Supplement: Supplement 1. — eTable 1. Preoperative biopsy characteristics eTable 2. Over staging and under staging of final pathological T staging by multiparametric MRI and 18F-PSMA-1007 PET/CT eTable 3. Diagnostic accuracy of 18F-PSMA-1007 PET/CT and MRI for lymph node metastasis eTable 4. Nodule level detection by multiparametric MRI and 18F-PSMA-1007 PET/CT Stratified by Gleason grade group of individual prostate cancer nodules [file jamaoncol-e243196-s001.pdf]

## Supplemental Online Content

Mookerji N, Pfanner T, Hui A, et al. Fluorine-18 prostate-specific membrane antigen PET/CT vs multiparametric MRI for locoregional staging of prostate cancer. *JAMA Oncol*. Published online July 1, 2024. doi:10.1001/jamaoncol.2024.3196

eTable 1. Preoperative biopsy characteristics

eTable 2. Over staging and under staging of final pathological T staging by multiparametric MRI and $^{18}$F-PSMA-1007 PET/CT

eTable 3. Diagnostic accuracy of $^{18}$F-PSMA-1007 PET/CT and MRI for lymph node metastasis

eTable 4. Nodule level detection by multiparametric MRI and $^{18}$F-PSMA-1007 PET/CT Stratified by Gleason grade group of individual prostate cancer nodules

This supplemental material has been provided by the authors to give readers additional information about their work.

© 2024 Mookerji N et al. *JAMA Oncology.*

**eTable 1: Preoperative Biopsy characteristics.**

| Characteristic | Results |
| --- | --- |
| **Biopsy Gleason Group, n (%)** | GG 1 – 4 (3)<br>GG 2 – 95 (70)<br>GG 3 – 24 (19)<br>GG 4 – 7 (5)<br>GG 5 – 4 (3) |
| **Number of Positive Cores, median (IQR)** | 6 (4-9) |
| **Total Biopsy Cores, median (IQR)** | 14 (12-15) |

© 2024 Mookerji N et al. *JAMA Oncology.*

**eTable 2: Over Staging and Under Staging of Final Pathological T Staging by Multiparametric MRI and $^{18}$F-PSMA-1007 PET/CT.**

| N=134 | MRI | PSMA PET/CT |
|---|---|---|
| **Correct staging, n (%)** | 38 (28) | 61 (45) |
| **Overestimated, n (%)** | 10 (7) | 18 (13) |
| **Underestimated, n (%)** | 86 (64) | 55 (41) |

© 2024 Mookerji N et al. *JAMA Oncology.*

**eTable 3: Diagnostic Accuracy of $^{18}$F-PSMA-1007 PET/CT and MRI for lymph node metastasis.**

| Parameter | MRI (95% CI) | PSMA PET/CT (95% CI) |
|---|---|---|
| **Lymph Node Metastasis** | | |
| **Sensitivity** | 25% (1-78) | 50% (9-91) |
| **Specificity** | 100% (96-100) | 98% (94-100) |
| **PPV** | 100% (5-100) | 50% (9-91) |
| **NPV** | 98% (93-99) | 98% (94-100) |

© 2024 Mookerji N et al. *JAMA Oncology.*

**eTable 4: Nodule Level Detection by Multiparametric MRI and $^{18}$F-PSMA-1007 PET/CT Stratified by Gleason Grade Group of Individual Prostate Cancer Nodules.**

| Gleason Grade Group | MRI | PSMA PET/CT | P Value |
|---|---|---|---|
| **1, n (%)** | 6/55 (11) | 16/55 (29) | 0.023 |
| **≥2, n (%)** | 143/231 (62) | 201/231 (87) | <0.001 |

© 2024 Mookerji N et al. *JAMA Oncology.*
