# Supplementary material for: Fluorine-18 Prostate-Specific Membrane Antigen–1007 PET/CT vs Multiparametric MRI for Locoregional Staging of Prostate Cancer
Source: JAMA Oncol. 2024 Jul 1;10(8):1097–103. doi: 10.1001/jamaoncol.2024.3196 (PMC11217889; doi:10.1001/jamaoncol.2024.3196)
Supplement: Supplement 2. — Data sharing statement [file jamaoncol-e243196-s002.pdf]

Data Sharing Statement

Mookerji. Fluorine-18 Prostate-Specific Membrane Antigen–1077 PET/CT vs Multiparametric MRI for Locoregional Staging of Prostate Cancer. *JAMA Oncol*. Published July 01, 2024. doi:10.1001/jamaoncol.2024.3196

Data
Data available: Yes
Data types: Deidentified participant data
How to access data: By request to authors
When available: With publication

Supporting Documents
Document types: None

Additional Information
Who can access the data: Researchers
Types of analyses: Aggregate deidentified data
Mechanisms of data availability: With a signed access agreement
